# Early encapsulation of peripancreatic fluid/necrosis collections on imaging (CECT) in acute pancreatitis: influential factors and clinical significance for prognosis

**DOI:** 10.1186/s12876-024-03145-7

**Published:** 2024-01-29

**Authors:** Ning Ning, Congyi Yu, Wenwu Sun, Yi Wen, Tongtian Ni, Huiqiu Sheng, Ying Chen, Li Ma, Erzhen Chen, Bing Zhao, Enqiang Mao

**Affiliations:** Department of Emergency Medicine, Ruijin Hospital, School of Medicine, Shanghai Jiao Tong University, Shanghai, 200025 P. R. China

**Keywords:** Acute pancreatitis, Contrast-enhanced computed tomography, Risk factors, Organ failure, Infection

## Abstract

**Background:**

To identify the factors influencing the early encapsulation of peripancreatic fluid/necrosis collections via contrast-enhanced computed tomography (CECT) and to determine the clinical significance of early encapsulation for determining the prognosis of acute pancreatitis (AP) patients.

**Methods:**

AP patients who underwent CECT between 4 and 10 days after disease onset were enrolled in this study. Early encapsulation was defined as a continuous enhancing wall around peripancreatic fluid/necrosis collections on CECT. Univariate and multivariate logistic regression analyses were performed to assess the associations between the variables and early encapsulation. Clinical outcomes were compared between the non-encapsulation and early encapsulation groups with 1:1 propensity score matching.

**Results:**

A total of 289 AP patients were enrolled. The intra-observer and inter-observer agreement were considered good (kappa statistics of 0.729 and 0.614, respectively) for identifying early encapsulation on CECT. The ratio of encapsulation increased with time, with a ratio of 12.5% on day 5 to 48.7% on day 9. Multivariate logistic regression analysis revealed that the longer time from onset to CECT examination (OR 1.55, 95% CI 1.23–1.97), high alanine aminotransferase level (OR 0.98, 95% CI 0.97–0.99), and high APACHE II score (OR 0.89, 95% CI 0.81–0.98) were found to be independent factors associated with delayed encapsulation. The incidence of persistent organ failure was significantly lower in the early encapsulation group after matching (22.4% vs 6.1%, *p* = 0.043). However, there was no difference in the incidence of infected pancreatic necrosis, surgical intervention, or in-hospital mortality.

**Conclusions:**

AP patients without early encapsulation of peripancreatic fluid/necrosis collections have a greater risk of persistent organ failure. In addition to longer time, the high APACHE II score and elevated alanine aminotransferase level are factors associated with delayed encapsulation.

## Introduction

Acute pancreatitis (AP) is a common gastrointestinal disease with a global annual incidence of 34 per 100,000 person-years [1, 2]. The majority of patients present with a mild clinical course, but approximately 20% of patients develop organ failure or peripancreatic infection. In these patients, the overall mortality rate remains as high as 20%-40% [3, 4].

Contrast-enhanced computed tomography (CECT) is highly sensitive and accurate for both diagnosing and evaluating the severity of acute pancreatitis. In the early phase, the use of CECT is strongly recommended within 4 to 10 days after symptom onset for the evaluation of pancreatic necrosis [5–7]. In the late phase (> 4 weeks), an enhancing encapsulated wall around peripancreatic fluid/necrosis collections on CECT is recognized as a sign of maturation and termed pseudocyst/walled off necrosis [6]. A well-defined enhancing wall defines the area of peripancreatic fluid/necrosis collections and is an important sign of surgical intervention in AP patients. Unfortunately, there is scarce evidence that detailed the timing of continuous wall formation on CECT or its clinical importance.

Thus, the aim of this study was to identify the influential factors associated with early encapsulation, as well as the prognostic significance of these imaging signs.

## Methods

### Patients

Consecutive adult patients (aged ≥ 18 years) diagnosed with AP according to the revised 2012 Atlanta guidelines [6] who were admitted to the Ruijin Hospital, Shanghai Jiaotong University School of Medicine between January 2019 and May 2022 were enrolled in this study. The patient exclusion criteria were as follows: (1) diagnosed with mild acute pancreatitis; (2) discharged or died before CECT; (3) had a history of AP, chronic pancreatitis or pancreatic malignancy; and (4) lacked peripancreatic fluid/necrosis collections on CECT.

### Data collection

The clinical variables were extracted from the electronic database for each patient. The baseline demographic information included age, gender, body mass index, comorbidities, etiology, and time from onset to CECT examination. Laboratory indicators include blood amylase, white blood cell count, platelet count, C-reactive protein, procalcitonin, lactate, alanine aminotransferase, pro-albumin, albumin, total bilirubin, creatinine, fibrinogen, and D-dimers. Acute Physiology and Chronic Health Evaluation II (APACHE II) scores were collected within 24 h after admission. The CT severity index (CTSI) [8] and presence of pancreatic necrosis were assessed via CECT.

Clinical outcomes included the incidence of infected pancreatic necrosis; surgical intervention (percutaneous drainage, video assisted retroperitoneal debridement or open abdominal debridement); persistent organ failure (cardiovascular, respiratory or renal failure persisting for more than 48 h, evaluated according to the modified Marshall scoring system [6]); and in-hospital mortality. Infected pancreatic necrosis is diagnosed according the bubble sign on CT scan, or the culture results of the peripancreatic collections. The indications for surgical interventions include suspicion of infection; on-going gastric outlet, biliary, or intestinal obstruction due to a large walled off necrotic collection or pseudocyst; and disconnected duct syndrome.

### Imaging analysis

**Fig. 1:**
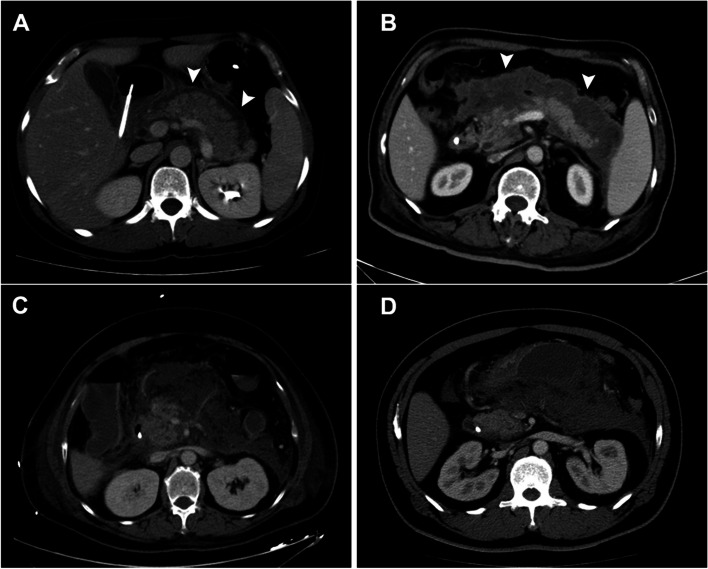
Axial contrast-enhanced CT scans in acute pancreatitis patients. **A**, **B** with encapsulation; **C**, **D**: without encapsulation. **A** 52-year-old woman on day 5 after symptom onset; (**B**) 71-year-old man on day 9 after symptom onset; (**C**) 59-year-old woman on day 8 after symptom onset; (**D**) 49-year-old man on day 9 after symptom onset. Arrowheads denote the encapsulation around the peripancreatic fluid/necrosis collections

CT data were acquired using two multidetector imaging machines (Somatom Perspective, Siemens, Germany and Optima CT 540, GE, USA), and the slice thickness was 3–5 mm. Nonionic intravenous contrast material was injected at a bolus of 3–5 mL/s with a total volume of 100–120 ml before scanning. All patients underwent unenhanced imaging followed by arterial phase (25–30 s) and venous phase (60 s) imaging after infusion of contrast material [9]. Encapsulation was defined as a continuous enhancing wall around peripancreatic fluid/necrosis collections on CECT **(**Fig. 1**)**. All the CT images were independently assessed by two specialists with 5 years of experience in AP management who were blinded to the clinical data.

### Data statistics

Categorical data will be described as the frequency or ratio. Continuous variables will be described using medians and interquartile ranges (IQRs). Categorical variables were compared using the χ2 test or Fisher’s exact test. Continuous variables were compared using the t test for normally distributed variables or the Wilcoxon rank-sum test for nonnormally distributed variables. We investigated potential influential factors for early encapsulation by using univariate and multivariate logistic regression. We compared the clinical results of the non-encapsulation group and early encapsulation group with 1:1 propensity score matching (PSM) using the nearest neighbor approach with no replacement and a caliper of 0.05. The variables used for matching were independent factors according to multivariate logistic regression. We used kappa statistics for intra-observer and inter-observer agreement for assessing early encapsulation on CECT. A kappa statistic of 0.41–0.60 was considered moderate agreement, 0.61–0.80 was considered good agreement, and 0.81–1.00 was considered excellent agreement. A two-sided *p* value < 0.05 was considered to indicate statistical significance. All the statistical analyses were performed using R software (version 4.2.1).

## Results

### Data screening

**Fig. 2:**
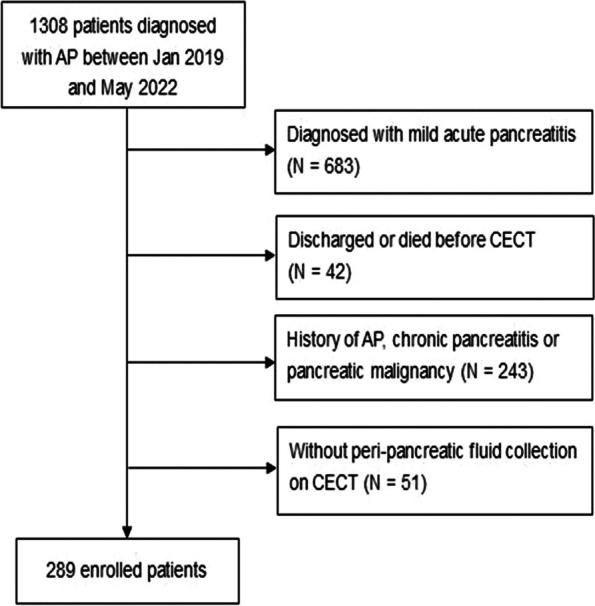
Flow diagram of participant selection in the study. AP: acute pancreatitis; CECT: contrast-enhanced computed tomography

A total of 1308 patients diagnosed with AP between Jan 2019 and May 2022 were enrolled. A total of 1019 patients were excluded based on the exclusion criteria. The data of the remaining 289 patients who underwent CECT between 4 and 10 days after symptom onset were included in the analysis (Fig. 2). 80 (28.6%) patients presented with a continuous enhancing wall (early encapsulation) around the peripancreatic fluid/necrosis collections on CECT. The intra-observer and inter-observer agreement were considered good, with kappa statistics of 0.729 and 0.614, respectively.

### Baseline characteristics and factors associated with early encapsulation in acute pancreatitis

**Fig. 3:**
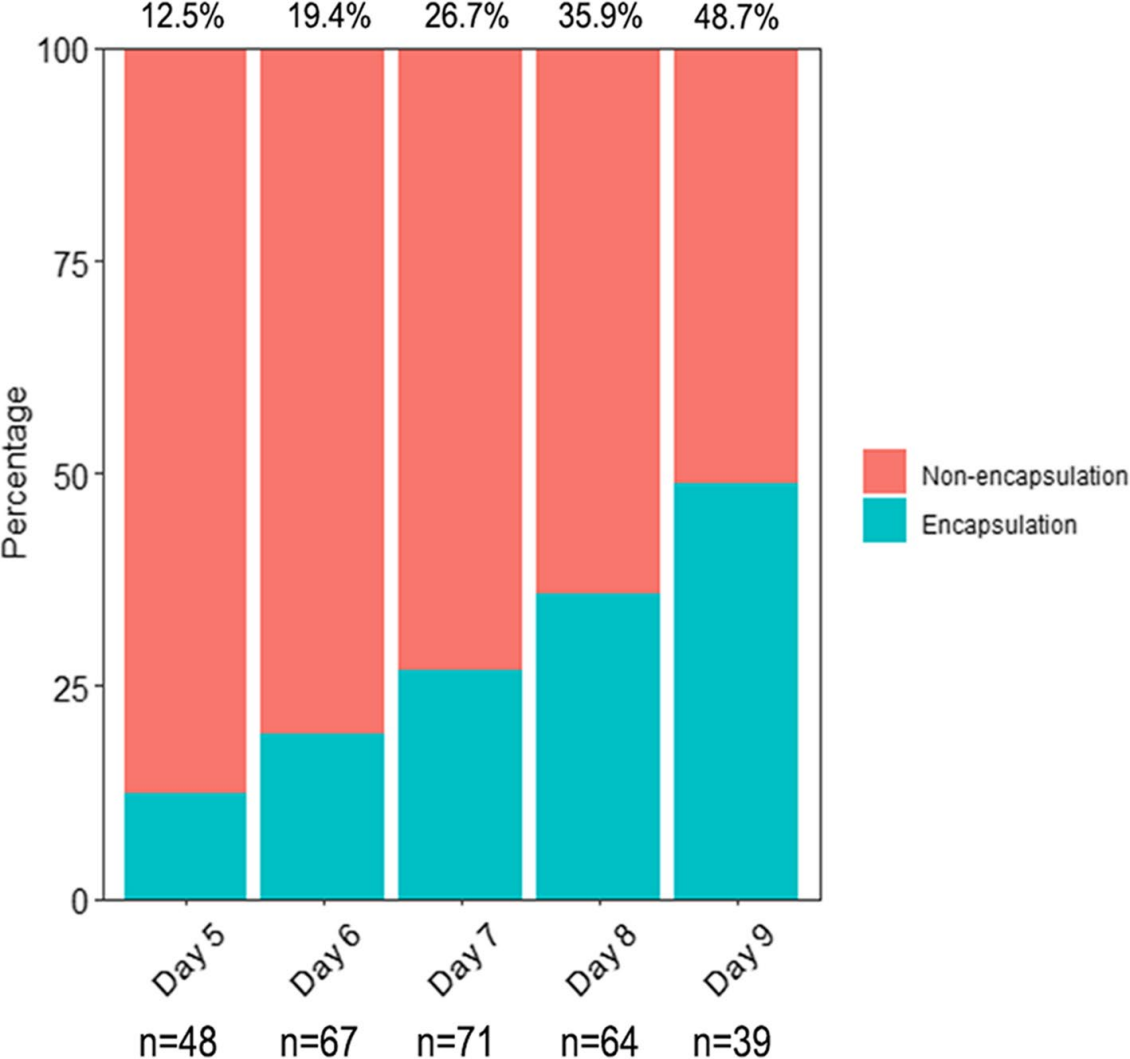
The percentage of early encapsulation on CECT at the given day. CECT: contrast-enhanced computed tomography

There were 48 (16.6%), 67 (23.2%), 71 (24.6%), 64 (22.1%) and 39 (13.5%) patients underwent CECT imaging from day 5 to day 9, respectively. The ratio of encapsulation on each day increased with time, with a ratio of 12.5% on day 5 to 48.7% on day 9 (Fig. 3). Among the 289 enrolled patients, the median age was 42 years, and 64% were males. There was no significant difference between the two groups’ demographic data (age, gender, body mass index, comorbidity, or etiology). Most of the laboratory indices at admission were comparable between the two groups, except for C-reactive protein, alanine aminotransferase, total bilirubin and creatinine. The CTSI score and incidence of pancreatic necrosis were comparable between the two groups, but the APACHE II score at admission was significantly greater in non-encapsulation group (median 8 vs 8, *p* = 0.012) **(**Table 1).

**Table 1:** Baseline characteristics of patients

Variables	Total (*N* = 289)	Non-encapsulation (*N* = 209)	Early encapsulation (*N* = 80)	*p* value
Demographic				
Age (years)	45.0(35.0–57.0)	43.0(35.0–57.0)	47.0(39.0–55.0)	0.645
Gender (male%)	64.0(185/289)	67.0(140/209)	56.2(45/80)	0.089
Body mass index (kg/m2)	25.7(23.4–28.4)	26.1(24.0–29.3)	24.1(22.7–26.0)	0.173
Comorbidity (%)				
Diabetes	30.4(88/289)	32.1(67/209)	26.2(21/80)	0.337
Hypertension	30.8(89/289)	33.5(70/209)	23.8(19/80)	0.108
Etiology (%)				
Biliary	40.8(118/289)	41.6(87/209)	38.8(31/80)	0.656
Hypertriglyceridemia	41.5(120/289)	41.6(87/209)	41.2(33/80)	0.954
Alcoholic	7.6(22/289)	7.2(15/209)	8.8(7/80)	0.652
others	10.0(29/289)	9.6(20/209)	11.2(9/80)	0.671
Time from onset to CECT examination (day)	7.3(6.4–8.4)	7.0(6.1–8.1)	8.0(7.0–8.6)	< 0.001*
Laboratory indicator				
Blood amylase (U/L)	524.0(222.5–1090.5)	518.5(228.2–1084.2)	599.0(115.0–1144.0)	0.584
White blood cell (× 109/L)	11.6(8.7–15.4)	11.6(8.9–15.4)	11.6(7.8–15.2)	0.190
Platelet (× 109/L)	174.0(131.0–211.0)	171.0(123.0–211.0)	185.5(139.8–218.2)	0.208
C-reactive protein (mg/L)	247.0(155.0–310.2)	259.0(162.8–317.0)	207.4(152.0–285.0)	0.033*
Procalcitonin (ng/mL)	2.0(0.8–4.9)	2.5(1.0–5.8)	0.9(0.5–2.0)	0.059
Lactate (mmol/L)	1.9(1.4–2.8)	1.9(1.5–2.9)	1.9(1.3–2.7)	0.888
Alanine aminotransferase (U/L)	21.0(14.0–42.0)	23.5(16.0–49.2)	17.5(13.0–28.5)	0.017*
Pro-albumin (g/L)	123.5(85.0–166.5)	122.0(85.0–161.2)	132.0(89.5–179.8)	0.055
Albumin (g/L)	32.0(29.0–35.0)	33.0(29.0–35.0)	32.0(29.5–35.5)	0.958
Total bilirubin (μmol/L)	21.5(15.3–31.2)	23.1(16.6–32.2)	17.7(12.1–24.8)	0.037*
Creatinine (μmol/L)	67.0(54.0–87.0)	71.0(55.8–96.2)	58.0(48.0–69.0)	0.004*
Fibrinogen (g/L)	5.8(4.4–6.7)	5.8(4.4–6.8)	5.6(4.5–6.5)	0.834
D-dimers (mg/L)	4.7(3.1–7.5)	4.9(3.3–8.5)	4.6(2.7–6.8)	0.090
Clinical scoring				
APACHEII score	8(6–11)	8(6–11)	8(5–9)	0.012*
CTSI score	6(4–6)	6(4–8)	6(5–6)	0.154
Pancreatic necrosis	72.3(209/289)	70.8(148/209)	76.2(61/80)	0.355
Outcomes				
Infected pancreatic necrosis (%)	12.5(36/289)	14.8(31/209)	6.2(5/80)	0.075
Surgical intervention (%)	13.8(40/289)	16.3(34/209)	7.5(6/80)	0.053
Persistent organ failure(%)	25.3(73/289)	31.6(66/209)	8.8(7/80)	< 0.001*
In-hospital mortality(%)	4.5(13/289)	5.7(12/209)	1.2(1/80)	0.183

The data are presented as percentages (numbers) for categorical data and medians (IQRs) for continuous data

*APACHE II* Acute Physiology and Chronic Health Evaluation II, *CTSI* CT severity index

^*^p < 0.05

Univariate logistic regression analysis identified several factors associated with early encapsulation in acute pancreatitis, including the time from onset to CECT examination, C-reactive protein level, alanine aminotransferase level, total bilirubin, creatinine level, and APACHE II score. We selected age, gender, body mass index, and statistically significant factors for multivariate logistic regression. We found that the longer time from onset to CECT examination (OR 1.55, 95% CI 1.23–1.97), high alanine aminotransferase level (OR 0.98, 95% CI 0.97–0.99), and high APACHE II score (OR 0.89, 95% CI 0.81–0.98) were independent factors associated with delayed encapsulation in acute pancreatitis after adjustment for confounders (Table 2).

**Table 2:** Univariate logistic regression analysis and multivariate logistic regression analysis of factors associated with early encapsulation

**Factors**	**Univariable Analysis**		**Multivariable Analysis**	
	**OR (95% CI)**	***p* value**	**OR (95% CI)**	***p* value**
Age (years)	1.00 (0.98–1.02)	0.64		
Gender (male vs female)	0.63 (0.37–1.07)	0.09		
Body mass index (kg/m2)	1.00 (0.99–1.01)	0.85		
Time from onset to CECT examination (day)	1.58(1.27–1.96)	< 0.001*	1.55(1.23–1.97)	< 0.001*
C-reactive protein (mg/L)	0.99 (0.99–1.00)	0.032*		
Procalcitonin (ng/mL)	0.96 (0.92–1.00)	0.086		
Alanine aminotransferase (U/L)	0.98 (0.97–0.99)	0.012*	0.98(0.97–0.99)	0.04*
Pro-albumin (g/L)	1.00 (0.99–1.00)	0.061		
Total bilirubin (μmol/L)	0.96 (0.94–0.99)	0.005*		
Creatinine (μmol/L)	0.98 (0.97–0.99)	0.005*		
D-dimers (mg/L)	0.95 (0.90–1.01)	0.15		
APACHEII score	0.89 (0.83–0.96)	0.004*	0.89(0.81–0.98)	0.02*

*OR* odds ratio, *APACHE II* Acute Physiology and Chronic Health Evaluation II, *CECT* contrast-enhanced computed tomography

^*^p < 0.05

### Differences in patient outcomes between the non-encapsulation group and early encapsulation group after propensity score matching

After matching for the variables of time from onset to CECT examination, alanine aminotransferase level, and APACHE II score, 98 patients were included in the final analysis. The characteristics of the two groups were comparable after matching. The clinical outcome analysis revealed no significant differences in the incidence of infected pancreatic necrosis (4.1% vs 8.2%, *p* = 0.673), surgical intervention (6.1% vs 10.2%, *p* = 0.712), or in-hospital mortality (2% vs 0%, *p* = 1) between the two groups. However, the incidence of persistent organ failure was significantly lower in the early encapsulation group (22.4% vs 6.1%, *p* = 0.043) (Table 3).

**Table 3:** Baseline characteristics and outcomes between the encapsulation group and early encapsulation group after propensity score matching

Variables	Non-encapsulation (*N* = 49)	Early encapsulation (*N* = 49)	*p* value
Demographic			
Age (years)	43.47 (16.61)	47.18 (14.26)	0.238
Gender (male%)	59.2 (29/49)	46.9 (23/49)	0.312
Body mass index (kg/m2)	26.92 (4.10)	31.70 (47.59)	0.485
Time from onset to CECT examination (day)	7.41 (1.22)	7.54 (1.30)	0.614
Laboratory indicator			
C-reactive protein (mg/L)	228.42 (102.94)	209.26 (96.75)	0.345
Lactate (mmol/L)	2.22 (0.96)	2.24 (1.72)	0.935
Alanine aminotransferase (U/L)	28.88 (27.64)	28.71 (31.93)	0.978
Total bilirubin (μmol/L)	36.13 (74.71)	21.97 (15.91)	0.198
Creatinine (μmol/L)	83.20 (86.87)	64.31 (32.52)	0.157
Clinical scoring			
APACHEII score	7.61 (3.70)	7.96 (3.10)	0.616
CTSI score	5.47 (1.67)	5.67 (1.53)	0.530
Outcomes			
Infected pancreatic necrosis (%)	4.1 (2/49)	8.2 (4/49)	0.673
Surgical intervention (%)	6.1 (3/49)	10.2 (5/49)	0.712
Persistent organ failure(%)	22.4 (11/49)	6.1 (3/49)	0.043*
In-hospital mortality(%)	2.0 (1/49)	0 (0/49)	1.000

The data are presented as percentages (numbers) for categorical data and means (SDs) for continuous data

*APACHE II* Acute Physiology and Chronic Health Evaluation II, *CTSI* CT severity index

^*^p < 0.05

## Discussion

According to the current study of 289 AP patients, those who did not have their peripancreatic fluid/necrosis collections encapsulated between 4 and 10 days after disease onset had a greater risk of persistent organ failure. In addition to the longer time, we found that high alanine aminotransferase levels and APACHE II score at admission were factors associated with delayed encapsulation.

According to the revised 2012 Atlanta guidelines, in mild AP, there are only some inflammatory changes in the pancreas and peripancreatic fat on CT. In more severe AP, CT scans often reveal peripancreatic fluid collections with or without pancreatic necrosis [6]. Peripancreatic fluid collections contain many high-molecular-weight cytokines, proteases, and unsaturated fatty acids, which can induce continuous inflammatory reactions and worsen outcomes [10, 11]. The formation of fibrous and granulated tissue is a way that the body repairs injury and restricts damage. The necrotic tissue and fluid are organized and absorbed after encapsulation. In AP, this fibrous and granulated tissue is shown as a contrast-enhancing wall around the peripancreatic fluid/necrosis collections on CECT. The wall becomes obvious on CT image over time. Four weeks after disease onset, the wall defined the extent of the pseudocyst and walled-off the pancreatic necrosis. However, at this stage, the wall is thick and already “mature”. There was a cohort study from the Netherlands which described the natural history of encapsulation of peripancreatic collections [12]. In this study, encapsulation was classifies as medium, largely and fully encapsulation according to the degree of encapsulation. Medium encapsulation represents the early encapsulation. It was found that medium encapsulation was seen in 11% patients in the first week, and in 56% patients in the second week. In our study, we report that as early as day 5 after onset, the wall occurs in 12.5% of AP patients, and the percentage of cases increases to 48.7% on day 9. The percentage reported in our study is consistent with previous reports.

According to current AP management guidelines, CECT is not recommended within 3 days after onset but is strongly recommended within 10 days after onset for severity assessment [5, 13]. CECT provides crucial information for severity prediction in AP patients. Two important prognostic scoring systems—the CTSI and modified CTSI—are based on CECT. It has been reported that the CTSI score and modified CTSI score more accurately diagnose clinical severity than do the other clinical signs and laboratory indicator-based scoring systems [14, 15]. Generally, the most concerning issues on CECT are pancreatic necrosis, peripancreatic inflammation, and extrapancreatic complications.

In recent years, other imaging indices have also been proposed to be useful predictive factors of severity. In the study of Meyrignac et al., the extrapancreatic necrosis volume provided more reliable information for predicting organ failure and infection than did the current scoring systems [16]. Another study concluded that the volume and mean CT density of necrotic tissue based on CECT help with the early prediction of organ failure [17]. However, the professional software needed for analysis limits the clinical generalization of these new imaging indicators. It is well known that enhancing walls around fluid/necrosis collections on CECT can be easily observed at the middle and later stages of AP. In our study, the enhancing wall could also be observed at an early stage by specialists in AP management, with good intra-observer and inter-observer agreement (kappa statistics of 0.729 and 0.614, respectively).

The present study revealed that early encapsulation has no effect on the incidence of infected pancreatic necrosis or surgical intervention, neither before matching nor after matching. This result indicated that the encapsulated wall could not impede the translocation of bacteria. The formation of mature encapsulation is regarded as the ideal timing for surgical intervention [18], but our results showed that early encapsulation could not reduce the incidence of surgical intervention. In the past 10 years, step-up surgical or step-up endoscopic approach has been recommended [13]. In this study, radiological or ultrasound guided percutaneous drainage is the first choice for drainage, followed by video assisted retroperitoneal debridement or open abdominal debridement. It should be pointed out that, the low events number in each group could bias the statistical result. Evidence from large cohort study is needed. However, the incidence of persistent organ failure was significantly lower in the early encapsulation group (31.6% vs 8.8%, p < 0.001 before matching; 22.4% vs 6.1%, *p* = 0.043 after matching). The reason may be that fibrous and granulated tissue is a barrier that can effectively reduce the absorption of harmful inflammatory cytokines in peripancreatic collections. An important finding of our study is that, in addition to time, the occurrence of early encapsulation was independently associated with the APACHE II score and alanine aminotransferase level. A high APACHEII score and elevated alanine aminotransferase level are associated with the delay encapsulation of the peripancreatic collections. The underlying mechanism is unknown and needs to be explored in the future study.

Our study has several limitations. First, only patients with peripancreatic fluid/necrosis collections on CECT were included in the analysis, which introduces selection bias. However, patients without peripcreatic fluid/necrosis collections on CECT are classified as mild AP, and the prognosis is very satisfying. Second, the time from onset to CECT examination was not fixed in our study. However, we matched patients according to the time factor in the propensity score matching, which made the time factor comparable between the two groups. Third, due to spatial heterogeneity of the encapsulation, there is a lack of exact definition of early encapsulation. Two specialists independently assessed the CECT, and intra-observer and inter-observer agreement were reported with a promising result.

## Conclusion

In summary, AP patients without early encapsulation of peripancreatic fluid/necrosis collections on CECT have a greater risk of persistent organ failure. In addition to longer time, the high APACHE II score and elevated alanine aminotransferase level are factors associated with the likelihood of delayed encapsulation.

## Data Availability

The requests should be sent to the corresponding author, Enqiang Mao or Bing Zhao.

## Abbreviations

CECT: Contrast enhanced computed tomography
AP: Acute pancreatitis
APACHE II: Acute Physiology and Chronic Health Evaluation II
CTSI: CT severity index
IQR: Interquartile range
PSM: Propensity score matching
